# Human Immunoglobulin G Cannot Inhibit Fibrinogen Binding by the Genetically Diverse A Domain of *Staphylococcus aureus* Fibronectin-Binding Protein A

**DOI:** 10.1128/mSphere.00590-17

**Published:** 2018-03-07

**Authors:** P. Martijn den Reijer, Mehri Tavakol, Nicole Lemmens-den Toom, Dikra Allouch, Sheila Thomas, Vannakambadi K. Ganesh, Ya-Ping Ko, Henri A. Verbrugh, Willem J. B. van Wamel

**Affiliations:** a Department of Medical Microbiology and Infectious Diseases, Erasmus University Medical Center, Rotterdam, The Netherlands; b Center for Infectious and Inflammatory Diseases, Institute of Bioscience and Technology, Texas A&M Health Science Center, Houston, Texas, USA; University of Nebraska Medical Center

**Keywords:** *Staphylococcus aureus*, antibody function, antibody repertoire, bacteremia, fibrinogen, fibronectin-binding protein A, Luminex

## Abstract

The fibronectin-binding protein A (FnBPA) is a cell surface-associated protein of *Staphylococcus aureus* which mediates adherence to the host extracellular matrix and is important for bacterial virulence. Previously, substantial sequence diversity was found among strains in the fibrinogen-binding A domain of this protein, and 7 different isotypes were described. The effect of this sequence diversity on the human antibody response, in terms of both antibody production and antibody function, remains unclear. In this study, we identify five different FnBPA A domain isotypes based on the sequence results of 22 clinical *S. aureus* isolates, obtained from the same number of patients suffering from bacteremia. Using a bead-based Luminex technique, we measure the patients’ total immunoglobulin G (IgG) against the 7 FnBPA isotypes at the onset and during the time course of bacteremia (median of 10 serum samples per patient over a median of 35 days). A significant increase in IgG against the FnBPA A domain, including the isotype carried by the infecting strain, is observed in only three out of 22 patients (14%) after the onset of bacteremia. Using a Luminex-based FnBPA–fibrinogen-binding assay, we find that preincubation of recombinant FnBPA isotypes with IgG from diverse patients does not interfere with binding to fibrinogen. This observation is confirmed using an alternative Luminex-based assay and enzyme-linked immunosorbent assay (ELISA).

**IMPORTANCE** Despite the many *in vitro* and murine *in vivo* studies involving FnBPA, the actual presence of this virulence factor during human infection is less well established. Furthermore, it is currently unknown to what extent sequence variation in such a virulence factor affects the human antibody response and the ability of antibodies to interfere with FnBPA function. This study sheds new light on these issues. First, the uniform presence of a patient’s IgG against FnBPA indicates the presence and importance of this virulence factor during *S. aureus* pathogenesis. Second, the absence of an increase in antibody production in most patients following bacteremia indicates the complexity of *S. aureus*-host interactions, possibly involving immune evasion or lack of expression of FnBPA during invasive infection. Finally, we provide new insights into the inability of human antibodies to interfere with FnBPA-fibrinogen binding. These observations should be taken into account during the development of novel vaccination approaches.

## Abstract (precis)

Despite the many *in vitro* and murine *in vivo* studies involving FnBPA, the actual presence of this virulence factor during human infection is less well established. Furthermore, it is currently unknown to what extent sequence variation in such a virulence factor affects the human antibody response and the ability of antibodies to interfere with FnBPA function. This study sheds new light on these issues. First, the uniform presence of a patient’s IgG against FnBPA indicates the presence and importance of this virulence factor during *S. aureus* pathogenesis. Second, the absence of an increase in antibody production in most patients following bacteremia indicates the complexity of *S. aureus*-host interactions, possibly involving immune evasion or lack of expression of FnBPA during invasive infection. Finally, we provide new insights into the inability of human antibodies to interfere with FnBPA-fibrinogen binding. These observations should be taken into account during the development of novel vaccination approaches.

## INTRODUCTION

*Staphylococcus aureus* can both colonize human squamous epithelial surfaces (1, 2) and cause infections that are associated with significant morbidity and mortality, including bacteremia (3–5). The success of *S. aureus* in colonizing and infecting its human host is attributed to many virulence factors, including surface proteins such as the fibronectin-binding protein A (FnBPA) (6). This protein is encoded by the *fnbA* gene (7, 8) and is expressed by most strains of *S. aureus* (9). FnBPA has been shown to contribute to disease in diverse animal models of staphylococcal infection (10–12), and it has several functions. FnBPA promotes bacterial binding to fibrinogen, elastin, and fibronectin (13–15), which are glycoproteins that are present at various concentrations in the host extracellular matrix comprising, for instance, the mucosa and blood (16, 17). Binding between these molecules and FnBPA mediates bacterial adhesion to and invasion of endothelial and epithelial cells (18, 19), and it promotes biofilm formation (20, 21). The latter functions of FnBPA are considered to help *S. aureus* in evading antibiotics and the host immune response (18, 19, 22).

Binding of FnBPA to fibrinogen and elastin is mediated by its N-terminal A domain (15, 23), which consists of three separately folded N1, N2, and N3 subdomains that are structurally similar to the A domain of *S. aureus* clumping factor A (ClfA) and *Staphylococcus epidermidis* serine-aspartate (SD)-repeat-containing protein G (SdrG) (24, 25). The N2 and N3 subdomains of these surface proteins are separated by a hydrophobic trench, and residues lining this trench are especially important in binding to fibrinogen and elastin (23).

The important role of FnBPA in *S. aureus* pathogenesis has made it a potentially interesting target for vaccination approaches against this pathogen. Interference with the interactions between FnBPA and its substrates could potentially hamper bacterial colonization or infection of the host. However, although FnBPA has been successfully used as a vaccine target in animal models (19, 26, 27), it has yet to be tested in humans. Furthermore, despite promising results in animal models, targeting the functionally similar ClfA did previously not lead to a valid vaccine in humans (28–30). Several factors might complicate the successful use of FnBPA as a vaccine target, such as the (lack of) presence of FnBPA during invasive infection, which, despite the plethora of studies examining the role of FnBPA *in vitro* and in animal models *in vivo*, has been less well established *in vivo* in humans. Another factor might involve the substantial sequence diversity found in the FnBPA A domain (31), with seven different protein variants (isotypes) identified previously sharing only 66 to 76% amino acid identity (23, 32). Although all these isotypes bind fibrinogen with similar affinities, murine monoclonal antibodies raised against different isotypes exhibit only limited cross-reactivity (32). The effect of this sequence diversity on the human antibody response against FnBPA remains unclear and has not been taken into account in previous vaccination studies, including both animal models and clinical trials, nor in our previous studies characterizing patient antibody responses (33–35). Yet if such sequence diversity in FnBPA among clinical strains of *S. aureus* is present and would affect the human antibody response, this should be taken into account during the development of future vaccines.

It is currently also unknown whether natural antibodies against FnBPA, produced in patients after the onset of infection, can interfere with the binding between FnBPA and fibrinogen. Interestingly, it was previously shown that immunoglobulin G (IgG) obtained from patients with *S. aureus* infections did not interfere with the binding between fibronectin and recombinant FnBPA fragments or whole *S. aureus* cells, respectively (36). On the other hand, rabbit monoclonal antibodies against *S. aureus* FnBP fragments did inhibit fibronectin binding (37), and rabbit polyclonal antibodies raised against clumping factor B blocked binding to fibrinogen (38). Thus, the ability of naturally occurring antibodies to block the binding between *S. aureus* and constituents of the extracellular matrix remains uncertain.

The aims of this study were to further characterize sequence diversity in the A domain of FnBPA among 22 clinical strains isolated from the blood of the same number of patients suffering from bacteremia, to characterize the antibody responses against different isotypes in each patient, and to ascertain the ability of human antibodies to interfere with binding between FnBPA and fibrinogen.

(This work is part of the Ph.D. thesis of P. M. den Reijer.)

## RESULTS

### Variation of the FnBPA A domain in clinical *S. aureus* isolates.

We determined the prevalence of the seven known isotypes (I to VII) of the FnBPA A domain (23, 32) among 22 *S. aureus* strains isolated from the blood of patients suffering from bacteremia. The FnBPA A domain of each strain was sequenced, and genomic sequences were compared together with those of previously described reference strains for each isotype.

**FIG 1:**
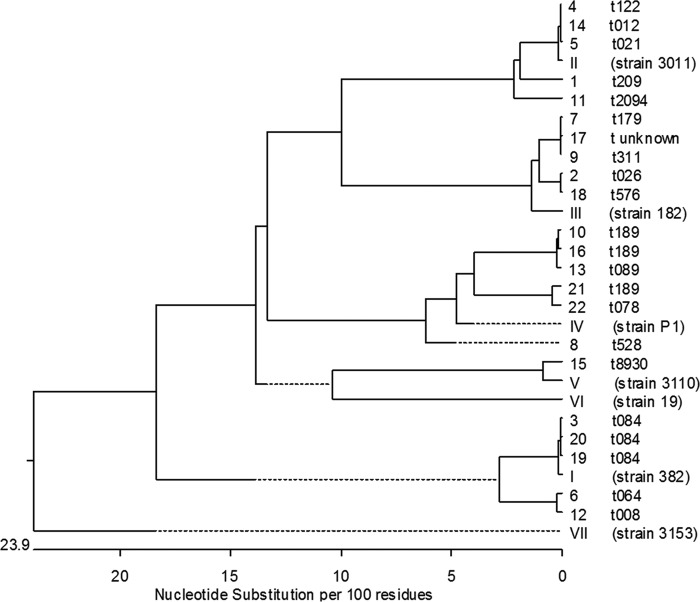
Genetic relationships of *fnbA* A domain between strains. *fnbA* gene segments encoding the entire FnBPA A domain were sequenced from 22 clinical isolates. Numbers indicate patient number, followed by the *spa* type of each strain. Sequences were aligned together with those of reference strains for the *fnbA* isotypes I to VII.

In line with previous results, substantial sequence diversity was found in the FnBPA A domain among *S. aureus* isolates (32). Five isotypes were identified among the 22 isolates: five strains encoded isotype I (92.7 to 100% amino acid identity to reference strain 382), five strains encoded isotype II (96 to 100% amino acid identity to reference strain 3011), five strains encoded isotype III (97.1 to 97.4% amino acid identity to reference strain 182), and six strains encoded isotype IV (91.9 to 97.1% amino acid identity to reference strain P1). One strain encoded isotype V (97.5% amino acid identity to reference strain 3110). The genetic relationships of the *fnbA* A domains of all strains, including the reference strains, are summarized in Fig. 1. Strains with the same *fnbA* isotype also shared similar *spa* types, while the single strain encoding isotype V had *spa* type t8930.

Since the isotype of the FnBPA A domain is primarily determined by the highly divergent N2N3 subdomains (32), recombinant proteins of these subdomains were expressed for each isotype. These proteins were covalently coupled to color-coded beads in a Luminex setup, allowing the simultaneous quantification of different antibodies directly in serum samples to characterize patient antibody responses against these variable subdomains.

### Characterization of IgG response against FnBPA isotypes in bacteremia patients.

**FIG 2:**
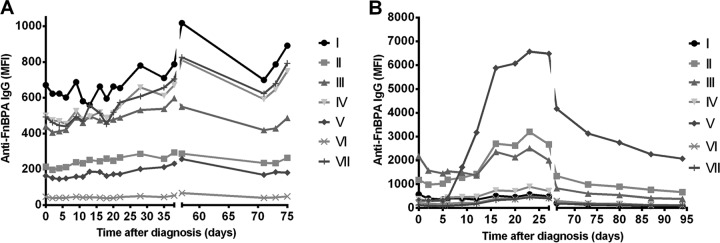
Course of IgG levels against FnBPA isotypes following bacteremia. (A) Representative course of IgG levels against 7 FnBPA isotypes (I to VII) following the onset of bacteremia (day 0, defined as the day of the first blood culture positive for *S. aureus*) in patient 1, infected with a strain carrying isotype II. (B) Same plot for patient 15, infected with a strain carrying isotype V. Data points represent the mean for two separate measurements per serum sample.

**FIG 3:**
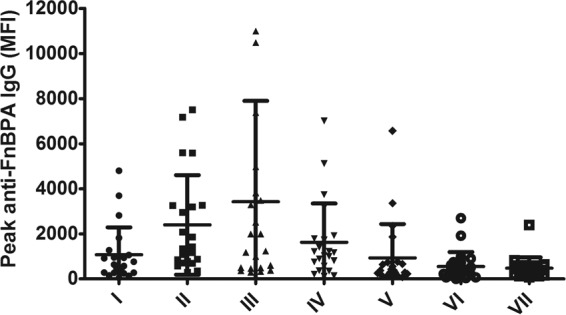
Variation in peak IgG levels against FnBPA isotypes after the onset of bacteremia. Scatter plot showing the distribution of peak IgG levels against all 7 FnbPA isotypes (I to VII) in 22 patients suffering from bacteremia. Each point represents one patient, and horizontal lines represent the medians and interquartile ranges.

Total IgG levels against all seven FnBPA A domain isotypes were prospectively measured in serum samples from patients after the onset of bacteremia (median of 10 serum samples per patient over a median of 35 days). Although patients were infected with a strain carrying one particular isotype, we observed that total IgG from each patient detectably bound all seven isotypes (Fig. 2). However, the height of IgG levels, especially against the more common isotypes I to IV and isotype V, varied considerably between patients (Fig. 3). This is in line with the heterogeneity observed previously in patient antibody responses after the onset of infection by our and other groups (33, 34, 39, 40).

In 18 out of 22 patients, the IgG levels directed against all isotypes did not significantly change after the onset of bacteremia (Fig. 2A; see Table S1 in the supplemental material), and we could not identify a specific increase in IgG against the isotype carried by the infecting strain. IgG levels against FnBPA did increase in the remaining four patients after the onset of bacteremia (patients 4, 15, 17, and 20 [Table S1]). In two of these patients, IgG levels against multiple isotypes (patient 17) or against another isotype than that of the infecting strain (patient 20) predominantly increased. Interestingly, in the remaining two patients (patients 4 and 15) a significant, more-than-20-fold increase in IgG levels predominantly against the isotype of the infecting strain was observed (Fig. 2B; Table S1).

10.1128/mSphere.00590-17.3 TABLE S1  Initial-to-peak fold increases in specific IgG levels for FnBPA isotypes in bacteremia patients. A fold increase of 1.0 means that the IgG level did not change during the course of infection compared to that of the initial measurement at the onset of bacteremia, whereas a fold increase of, e.g., 23.1 means that the peak IgG level in that patient after onset of infection was 23.1 times higher than the initial measurement. The FnBPA isotype of the infecting *S. aureus* strain, as determined with sequencing, is indicated in the second column. Significant initial-to-peak increases in IgG of more than 4-fold are indicated in italic boldface. Download TABLE S1, DOCX file, 0.01 MB. Copyright © 2018 den Reijer et al. 2018 den Reijer et al. This content is distributed under the terms of the Creative Commons Attribution 4.0 International license.

### FnBPA isotypes bind human fibrinogen.

To ascertain the ability of human antibodies, purified from the patients described above, to interfere with binding between FnBPA and fibrinogen, we developed a Luminex-based FnBPA–fibrinogen-binding assay. After covalently coupling recombinant FnBPA isotype proteins to beads, we confirmed that all seven isotypes were able to bind R-phycoerythrin (R-PE)-labeled human fibrinogen in a dose-dependent and saturable manner (Fig. S1A). This binding was further confirmed by a competition assay using R-PE-labeled and unlabeled fibrinogen, showing that the amount of bound PE-labeled fibrinogen decreased at increasing concentrations of unlabeled fibrinogen (Fig. S1B). Specific binding of fibrinogen by the FnBPA isotypes was confirmed by the lack of fibrinogen binding observed for the SD-repeat-containing protein E (SdrE) and iron-responsive surface determinant A (IsdA), which have low to no affinity for fibrinogen (41).

10.1128/mSphere.00590-17.1 FIG S1  Validation of fibrinogen binding by FnBPA isotypes. (A) Increasing concentrations of PE-labeled fibrinogen bind to all FnBPA isotypes but not to the control proteins (IsdA, SdrE, and ClfB) in a dose-dependent and saturable manner. (B) Adding increasing concentrations of unlabeled fibrinogen to a fixed amount of 1.5 μg of PE-labeled fibrinogen shows competition for the binding to all FnBPA isotypes. Data points represent the means for two separate measurements. Download FIG S1, TIF file, 0.4 MB. Copyright © 2018 den Reijer et al. 2018 den Reijer et al. This content is distributed under the terms of the Creative Commons Attribution 4.0 International license.

### Human IgG does not inhibit binding between FnBPA isotypes and fibrinogen.

Next, we investigated if purified IgG, isolated from bacteremia patients, could interfere with the binding between the FnBPA isotypes and fibrinogen. We confirmed that total IgG purified from five different patients, each infected with a strain carrying a different FnBPA isotype, and from polyclonal human IgG (PHG), obtained from noninfected volunteers, bound FnBPA isotypes I to V in a specific and dose-dependent manner (Fig. S2A and B). Human IgG did not notably bind to fibrinogen (data not shown).

10.1128/mSphere.00590-17.2 FIG S2  Specific and dose-dependent binding of human IgG to FnBPA isotypes I to V. (A) Beads coated with recombinant FnBPA isotypes I to V were incubated in a Luminex setup with a dilution range of total IgG purified from polyclonal human IgG (PHG) or patients (p14 to p19; for each FnBPA isotype, serum was chosen from a patient who was infected with a strain carrying that isotype). (B) FnBPA isotypes applied as a coating onto wells (0.5 µg/well) were incubated in an alternative ELISA setup with a dilution range of the same IgG, purified from PHG or patients. A plateau in antibody binding at higher concentrations can be observed, indicating that only FnBPA-specific IgG within the polyclonal antibodies binds. Download FIG S2, TIF file, 0.4 MB. Copyright © 2018 den Reijer et al. 2018 den Reijer et al. This content is distributed under the terms of the Creative Commons Attribution 4.0 International license.

**FIG 4:**
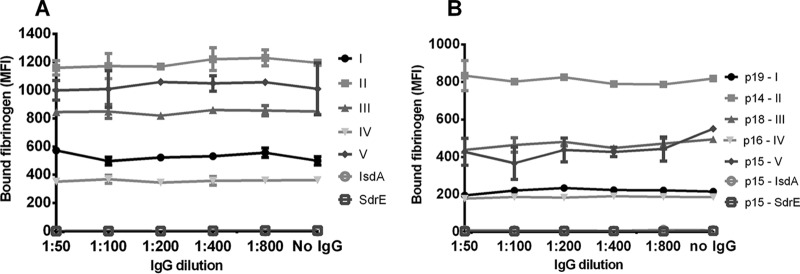
Preincubation of FnBPA isotypes with human IgG does not prevent binding of fibrinogen. (A) The most common isotypes, I to V, coupled to beads, were preincubated in this Luminex assay with a dilution range of IgG purified from polyclonal human IgG (PHG). Binding of a fixed concentration of PE-labeled fibrinogen was then measured. (B) Results of the same experiment using dilution ranges of IgG purified from different patients. Each isotype was preincubated with IgG from a patient who was infected with a strain carrying that isotype (e.g., patient 19 was infected with a strain carrying isotype I).

After preincubation of FnBPA isotypes I to V with the IgG from either PHG or patients, resulting FnBPA-bound IgG was allowed to bind PE-labeled fibrinogen. Based on results as shown in Fig. S1A, we chose a standard concentration of 1 μg PE-labeled fibrinogen per reaction mixture. Preincubation of FnBPA isotypes I to V with IgG from either PHG or patients did not interfere with the binding of this protein to PE-labeled fibrinogen, regardless of the IgG dilution (Fig. 4A and B). The amount of fibrinogen bound by the isotypes after preincubation with IgG dilutions did not significantly differ from controls without preincubation with IgG (one-way analysis of variance [ANOVA], significance level of *P* ≤ 0.05). Varying the starting concentration of fibrinogen did not affect these results, nor did simultaneous incubation of fibrinogen, IgG, and FnBPA isotypes (data not shown).

**FIG 5:**
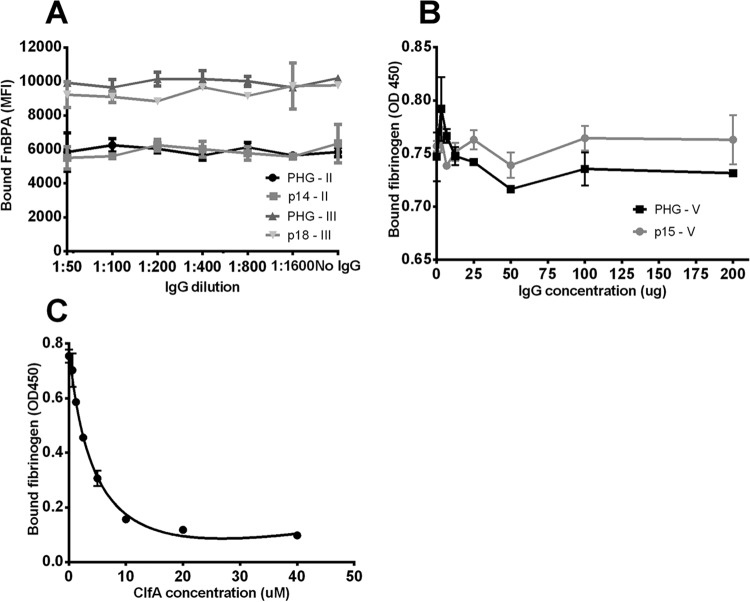
Confirmation of the inability of human IgG to prevent binding between FnBPA isotypes and fibrinogen. (A) PE-labeled, free FnBPA isotypes II and III were preincubated with a dilution range of total IgG either from PHG or from patients 14 and 18 (who were infected with strains carrying isotypes II and III, respectively). Protein-antibody complexes were then allowed to bind fibrinogen coupled to Luminex beads. (B) Using an alternative ELISA, preincubation of fibrinogen with IgG from patient 15 (infected with a strain carrying isotype V) did not clearly affect subsequent binding to coated FnBPA isotype V. Similar results were obtained for isotype I (data not shown). (C) Using the same ELISA, preincubation with ClfA, instead of IgG, inversely decreased the amount of remaining fibrinogen bound to FnBPA isotype V. All data points represent the mean for two measurements and are expressed as median fluorescence intensity (MFI) for panel A or as optical density at 450 nm for panels B and C.

To rule out the possibility that the coupling of FnBPA isotypes to the xMAP beads could somehow affect protein structure and thereby alter binding sites for IgG, which could alternatively explain the lack of interference by IgG as described above, we performed two alternative assays. In the first (Luminex-based) assay, fibrinogen coupled to beads was incubated with PE-labeled, free recombinant isotypes II and III. We confirmed a dose-dependent and saturable binding between the free FnBPA isotypes and immobilized fibrinogen (data not shown). Subsequently, we preincubated the free isotypes II and III with IgG from PHG or from one of the patients, followed by incubation with the immobilized fibrinogen. In line with the results described above, fibrinogen binding by both isotypes was not inhibited by any dilution of IgG (Fig. 5A). The amount of bound PE-labeled FnBPA after preincubation with IgG did not significantly differ from controls without preincubation with IgG (one-way ANOVA, significance level of *P* ≤ 0.05).

The results described above were further confirmed using enzyme-linked immunosorbent assay (ELISA), wherein isotypes I and V were immobilized onto 96-well plates and incubated with fibrinogen. Again, preincubation of both isotypes with a concentration range of IgG from either PHG or patients did not affect the amount of bound fibrinogen (Fig. 5B). As a control, we incubated fibrinogen with an increasing concentration of recombinant clumping factor A (ClfA) of *S. aureus*, which also binds to the same site on the fibrinogen r-chain as FnBPA, and measured the binding of fibrinogen to immobilized FnBPA. As expected, ClfA inhibited fibrinogen binding to FnBPA (Fig. 5C).

## DISCUSSION

We found that most patients suffering from serious invasive *S. aureus* infections do not respond to the microbial challenge by increasing production of preexisting IgG antibody titers directed against the A domain of the most common FnBPA isotypes, including the isotype carried by the infecting strain. Only 3 out of 22 patients (14%) mounted a significantly increased antibody response against FnBPA after the onset of bacteremia, which was predominantly directed against the isotype of the infecting strain in two patients. The immunoglobulins obtained from these and other patients, as well as from healthy volunteers, were not able to interfere with the binding between FnBPA and human fibrinogen *in vitro*.

In line with previous results, we found that *fnbA* isotypes I to IV were the most common among clinical isolates (32). Total IgG from patients could detectably bind these isotypes and was ubiquitously present at the onset of bacteremia. First, this ubiquitous presence of IgG against FnBPA suggests an important role of this virulence factor in the pathogenesis of *S. aureus*, presumably during colonization of the host and the early stages of infection. This is in line with the importance attributed to FnBPA during bacterial attachment, colonization, and infection in previously described *in vitro* and *in vivo* animal models (6, 11, 12).

Second, our results shed new light on the human antibody response toward FnBPA. Preexisting IgG levels that were detected at the onset of bacteremia against all isotypes—including that of the infecting strain—did not notably change during the course of infection in 18 out of 22 patients. This is in line with our previous results obtained with the same sera, showing that most virulence factors of *S. aureus* do not induce uniform increases in antibody titers after the onset of bacteremia (33). Notably, these patients were not immunosuppressed at the time of infection, and some virulence factors did induce a significant antibody response. Why other virulence factors, including FnBPA, do not uniformly induce a significant antibody response upon infection is currently unknown. Possibly, active manipulation of the host adaptive immune response by *S. aureus* plays a role (42, 43), or perhaps immunogenic epitopes of the FnBPA A domain are normally not exposed to the host adaptive immune system during infection due to the many interactions of this protein with host extracellular proteins (44). Finally, it could be that FnBPA is expressed only infrequently on the surface of *S. aureus* during the course of bacteremia, although *fnbA* mRNA was detectable during *in vitro* bacterial growth in human blood in our previous study (33). Interestingly, of the three patients who did mount a significant antibody response against FnBPA, one patient was infected with a strain carrying the rare isotype V. Although speculative, perhaps this isotype is associated with less-effective immune manipulation, more immunogenic exposure toward the host, and/or higher expression during bacteremia.

We currently do not know to what extent circulating antibodies differ in specificity for the different isotypes. On one hand, as opposed to the limited cross-reactivity observed previously for murine monoclonal antibodies raised against different isotypes (32), natural human polyclonal antibodies might possess cross-reactivity for different isotypes. This would be in line with our previous findings, wherein the same patient IgG was found to cross-react between the structurally similar leukocidin and gamma-hemolysin toxin components (33). Cross-reactivity could explain the simultaneous rise in IgG against multiple isotypes that was observed in two patients. On the other hand, the observation of *de novo* antibody production specifically against the isotype carried by the infecting strain in one patient suggests that specific, non-cross-reacting antibodies may be present as well.

The functionality of antibodies against FnBPA remains another subject of debate. Although data from antibodies raised in animal models suggest that they do block binding between *S. aureus* surface proteins and fibronectin (37, 38, 45), other studies, including those with human antibodies, do not show any interference with binding to fibronectin (36, 46). Our results are in line with the latter, demonstrating no apparent effect at any dilution of human IgG, purified from multiple patients, even from those with a significant antibody response against the isotype carried by the infecting strain, on binding between fibrinogen and FnBPA. A molecular explanation for this observation is currently lacking. Possibly, FnBPA binds with higher affinity to fibrinogen than to antibodies, or the two ligands have different binding sites on FnBPA. Alternatively, the functional binding domains within FnBPA remain immunologically hidden until the ligand is bound (36), or ligand binding might induce conformational changes leading to the exposition of other, nonfunctional binding sites for antibodies. The presence of these ligand-induced binding site (LIBS) antibodies against FnBPA has been demonstrated in patients suffering from *S. aureus* endocarditis (47). Finally, it has even been suggested that antibody binding might stabilize the interaction between FnBPA and fibrinogen (36), thereby actually enhancing binding, as observed for the binding of *Streptococcus dysgalactiae* FnBA to fibronectin when preincubated with mouse antibodies (46). Antibodies against FnBPA could have other functions than interfering with ligand binding. For instance, antibodies can assist in the opsonization of *S. aureus* (48) or in augmenting microbicidal killing by phagocytes (49).

We confirmed the inability of IgG to block FnBPA-fibrinogen binding with three alternative assays, to rule out that the coupling of either protein to xMAP beads could somehow affect correct antibody binding, for instance, due to conformational changes. Of note is that the measured median fluorescence intensities (MFIs) in the Luminex assay were much higher when free PE-labeled FnBPA isotypes were used instead of PE-labeled fibrinogen. Possibly, the large size of fibrinogen (50) would allow only a few PE-labeled molecules to bind to FnBPA-coated beads, while conversely, relatively many smaller PE-labeled FnBPA proteins could bind to fibrinogen-coated beads. Alternatively, the efficacies of coupling of the two proteins to beads might differ. We currently do not know what the efficacies of the coupling reactions are; however, even if coupling efficiencies differ, the interpretation of our inhibition experiments remains unchanged.

With regard to the suitability of FnBPA as a vaccine target, our results can be interpreted twofold. On one hand, we and others found that FnBPA-specific IgG is already circulating prior to the onset of clinical infection, probably resulting from multiple prior exposures to *S. aureus*, and yet this immune state does not confer protection from clinical disease (31, 33, 51, 52). Furthermore, the inability of human IgG to block binding between FnBPA and fibrinogen would argue against the use of FnBPA as a vaccine target. On the other hand, future vaccination approaches based on recombinant protein variants of FnBPA might induce novel, more protective antibodies against functionally important epitopes that are not normally targeted by the humoral immune response. This rationale is supported by previous work demonstrating that serum from unvaccinated human subjects was unable to block the interaction between *S. aureus* clumping factor A (ClfA) and fibrinogen, while antibodies elicited by a recombinant ClfA-containing vaccine could block this binding (53). Moreover, the level of functional antibody titers, able to block ClfA-fibrinogen binding *in vitro*, could directly be correlated with protection against *S. aureus* infection in an animal model *in vivo* (54). In this light, our results confirming the lack of functional antibodies against FnBPA in natural human sera, unable to protect against bacteremia, do not argue against the suitability of FnBPA as a vaccine target. In fact, our results might suggest that there is a window of opportunity for inducing novel, functionally interfering antibodies that can confer protection against invasive *S. aureus* infection.

To conclude, our results encourage further exploration into novel vaccination approaches that target the ubiquitously present FnBPA protein of *S. aureus*. Especially, further studies into the specific interactions between the FnBPA A domain and human antibodies, and their functional consequences, are needed.

## MATERIALS AND METHODS

### Ethics statement.

All patient serum samples used in this study were obtained from coded leftover material from routine diagnostic blood samples. In accordance with the guidelines of the Erasmus University Medical Center (MC) and the Dutch Federation of Biomedical Scientific Societies (Federatie van Medische Wetenschappelijke Verenigingen), all Erasmus MC patients are routinely informed of the possibility that leftover material from diagnostic samples may be used, albeit anonymously, for scientific research; all patients are routinely offered the opportunity to opt out in writing. Serum samples used in this study were obtained only from patients who did not object to the use of leftover material for scientific research and, in addition, gave verbal consent. This procedure was approved and the acquisition of additional written consent was waived specifically for this retrospective study by the Medical Ethics Committee of the Erasmus University Medical Center Rotterdam (MEC-2007-106, addendum 2). All serum samples were coded, and only qualified physicians of the Department of Medical Microbiology and Infectious Diseases had access to the original patient data.

### Bacterial strains and patients.

Serum samples and *S. aureus* isolates were obtained from 22 patients diagnosed with bacteremia, as described previously (33). In brief, bacteremia was defined as the isolation of *S. aureus* from at least one blood culture set. Starting from the first positive blood culture, a median number of 10 (interquartile range [IQR], 5 to 17) serum samples were collected per patient over a median period of 34.5 (interquartile range, 15 to 50) days.

### Sequencing of *fnbA* A domains.

DNA was isolated from strains using the QIAamp DNA minikit (Qiagen, Valencia, CA) according to the manufacturer’s protocol. The A domain of *fnbA* genes was amplified by PCR using previously described flanking primers (32) minus the restriction sites. Products resulting from amplifications were sequenced using a 3100 ABI Prism genetic analyzer (Applied Biosystems, Nieuwerkerk a/d IJssel, The Netherlands). Based on sequences of the flanking regions, individual primer sets were designed for each strain (sequences available upon request), and these were used to amplify and sequence the remaining part of each A domain. Overlapping sequences were merged for each strain and aligned with publicly available reference sequences of all FnBPA isotypes, which have been described previously (32). The complete sequences of all strains are available upon request. Primer design and alignments were performed using Primer-BLAST (available at https://www.ncbi.nlm.nih.gov) and MegAlign software (DNAStar Inc., Madison, WI, USA), respectively.

### Expression of recombinant FnBPA isotype proteins.

pQE30 constructs expressing His-tagged N2N3 A domains of isotypes I to VII were kindly provided by T. Foster (32). Each construct was verified by sequencing using pQE30-specific primers (sequence available upon request). Constructs were transformed into *Escherichia coli* Top 10, expressed by induction with isopropyl-β-d-1-thiogalactopyranoside (IPTG), and purified under denaturing conditions with nickel-nitrilotriacetic acid (Ni-NTA) agarose (Qiagen, Valencia, CA) according to the instructions of the manufacturer (QIAexpressionist handbook). Resulting proteins were quality controlled by SDS-PAGE and dialyzed against phosphate-buffered saline (PBS) for 24 h at 4°C.

### Measurement of antibodies.

IgG levels against all recombinant N2N3 A domains of FnBPA isotypes in the serum samples of bacteremia patients were measured using a bead-based flow cytometry technique (xMAP; Luminex Corporation, Austin, TX), as previously described (33). Briefly, recombinant proteins constituting the N2N3 A domains of FnBPA isotypes were covalently coupled to carboxylated xMAP beads, which were activated with *N*-hydroxysulfosuccinimide (sulfo-NHS) and 1-ethyl-3-(3-dimethylaminopropyl) carbodiimide (EDC) according to a previously optimized protocol (55). Protein-coated beads were then incubated with human serum which was diluted 1:100 in PBS. After repeated washing with PBS, bound antibodies were quantified using a 1:200 dilution of secondary phycoerythrin (PE)-labeled goat anti-human IgG antibodies. All measurements were performed in duplicate, and the median fluorescence intensities (MFIs), a semiquantitative measure of antibody levels, were averaged. Eighty-nine out of 1,827 duplicate measurements (0.05%) had coefficients of variation larger than 25% and were excluded from further analysis. All measurements were corrected for nonspecific background signal by subtracting the MFIs of control beads not coupled to any protein. The course of IgG levels over time was assessed graphically, and initial-to-peak increases in IgG levels were calculated for all isotypes in each patient. A more-than-4-fold initial-to-peak increase in IgG within 2 weeks after the onset of bacteremia was considered proof of a significant immune response.

### Luminex-based FnBPA–fibrinogen-binding assay.

Recombinant FnBPA isotypes and the non-fibrinogen-binding surface proteins SdrE and IsdA were coupled to xMAP beads as described above. Human fibrinogen (Calbiochem, Merck, Darmstadt, Germany) was labeled with phycoerythrin (PE) using the Lightning-Link R-PE conjugation kit (Innova Biosciences, Cambridge, United Kingdom) according to the manufacturer’s protocol, starting with 15 mg fibrinogen per 10 ml of 50 mM 2-(*N*-morpholino)-ethanesulfonic acid (MES) buffer. Protein-coated xMAP beads were incubated with various concentrations of PE-labeled fibrinogen, and after washing with PBS-1% bovine serum albumin (BSA), median fluorescence intensities (MFIs) were determined as a measure of bound fibrinogen using the same bead-based flow cytometry technique as described above. To study the effect of potentially interfering antibodies, total IgG was purified from sera of 5 different bacteremia patients and from pooled serum obtained from healthy volunteers (polyclonal human IgG [PHG]) using a HiTrap Protein G HP 1-ml column (GE Healthcare, Fairfield, CT) according to the manufacturer’s protocol. Purified IgG was dialyzed against PBS for 24 h at 4°C, and resulting antibody concentrations were 4.7 mg/ml for PHG and a median of 5.1 mg/ml (interquartile range [IQR], 4.4 to 8.8 mg/ml) for patient sera. Bead-bound isotypes were preincubated with a dilution range of the purified IgG, washed with PBS-1% BSA, and then incubated with a standard concentration of 1 μg PE-labeled fibrinogen per reaction mixture.

For the alternative Luminex-based binding assay, fibrinogen was coupled to beads and recombinant proteins of FnBPA isotypes II and III were PE labeled as described above. Free FnBPA isotypes were preincubated with purified IgG and then incubated with the fibrinogen-coated beads according to the protocol described above. All binding assays were repeated separately twice, and figures present the means for these measurements.

### ELISA-based FnBPA–fibrinogen-binding assay.

An enzyme-linked immunosorbent assay (ELISA) was performed to further confirm FnBPA-fibrinogen binding as previously described (56). In brief, 96-well Immulon 4HBX microtiter plates (Thermo Scientific, Waltham, MA) were coated overnight at 4°C and with gentle rotation with 0.5 μg per well of FnBPA isotypes, diluted in PBS. After blocking the wells with 2% BSA in PBS, plates were washed once with Tris-buffered saline (TBS) and coated isotypes were preincubated with a concentration range of purified IgG, diluted in TBS, for 1 h at room temperature. Subsequently, after 3 additional wash steps with TBS plus 0.1% Tween, plates were incubated for another hour with a fixed concentration of 40 nM fibrinogen (diluted in TBS). After another 3 wash steps, the bound fibrinogen was detected through incubation with horseradish peroxidase (HRP)-conjugated antifibrinogen antibodies (1:10,000 dilution) (Thermo Scientific) for 1 h and quantified after adding the substrate *o*-phenylenediamine dihydrochloride (OPD) (Sigma-Aldrich). The resulting absorbance at 450 nm (optical density [OD] at 450 nm) was measured in an ELISA microplate reader (Thermomax).

In the case of the ClfA competition assay, 40 nM fibrinogen was preincubated for 1 h with a 2-fold concentration range of recombinant ClfA (40 to 0.625 µM) and then incubated with FnBPA isotype-coated plates as described above.

### Statistical analysis.

Fold increases in antibody levels were calculated as the ratio of the peak antibody level divided by the initial antibody level (as measured in the first serum sample). One-way ANOVA with the duplicate measurements of bound PE-labeled fibrinogen (or FnBPA isotypes) as dependent variables and the IgG dilutions as factor were used to determine the significance of any differences in bound protein between different IgG dilutions. *P* values of ≤0.05 were considered statistically significant. IBM SPSS Statistics version 21 (IBM Corporation, Armonk, NY) was used for statistical analysis. Graphics were made using GraphPad Prism version 5 (GraphPad Inc., La Jolla, CA).
