# Supplementary material for: Human Immunoglobulin G Cannot Inhibit Fibrinogen Binding by the Genetically Diverse A Domain of Staphylococcus aureus Fibronectin-Binding Protein A
Source: mSphere. 2018 Mar 7;3(2):e00590-17. doi: 10.1128/mSphere.00590-17 (PMC5853482; doi:10.1128/mSphere.00590-17)
Supplement: TABLE S1 [file sph002182493st1.docx]

**Table S1.**

|  |  | Fold-increase in IgG specific for isotype | | | | | | |
| --- | --- | --- | --- | --- | --- | --- | --- | --- |
| Patient | Isotype infecting strain | Isotype I | Isotype II | Isotype III | Isotype IV | Isotype V | Isotype VI | Isotype VII |
| 1 | II | 1,7 | 1,4 | 1,4 | 1,6 | 1,6 | 1,4 | 1,5 |
| 2 | III | 2,8 | 2,7 | 3,7 | 2,7 | 1,0 | 1,0 | 1,0 |
| 3 | I | 1,5 | 3,8 | 1,4 | 1,4 | 1,5 | 1,5 | 1,3 |
| 4 | II | ***7,3*** | ***23,1*** | 3,8 | 1,9 | 2,3 | 2,1 | 2,0 |
| 5 | II | 1,1 | 1,0 | 1,0 | 1,1 | 1,0 | 1,0 | 1,0 |
| 6 | I | 1,7 | 2,4 | 2,3 | 2,7 | 2,9 | 2,9 | 3,1 |
| 7 | III | 1,8 | 1,6 | 1,7 | 1,5 | 1,6 | 1,7 | 1,6 |
| 8 | IV | 1,0 | 1,1 | 1,1 | 1,0 | 1,0 | 1,1 | 1,1 |
| 9 | III | 2,0 | 1,7 | 1,3 | 2,2 | 1,9 | 1,7 | 2,6 |
| 10 | IV | 1,0 | 1,1 | 1,0 | 2,9 | 1,4 | 1,4 | 2,9 |
| 11 | II | 2,0 | 2,7 | 2,2 | 3,1 | 3,3 | 2,1 | 2,0 |
| 12 | I | 1,4 | 1,0 | 1,1 | 1,0 | 1,2 | 1,1 | 1,4 |
| 13 | IV | 2,8 | 1,2 | 1,1 | 2,5 | 1,1 | 2,2 | 1,5 |
| 14 | II | 1,0 | 1,0 | 1,1 | 1,1 | 1,0 | 1,0 | 1,4 |
| 15 | V | 1,0 | 2,7 | 1,2 | 2,4 | ***20,6*** | 2,2 | 3,7 |
| 16 | IV | 1,0 | 1,0 | 1,2 | 1,0 | 1,0 | 1,0 | 1,0 |
| 17 | III | ***7,6*** | ***4,3*** | ***9,3*** | ***10,8*** | ***7,1*** | 3,6 | 1,6 |
| 18 | III | 1,5 | 2,1 | 1,7 | 1,5 | 2,0 | 2,0 | 2,6 |
| 19 | I | 1,2 | 1,5 | 1,1 | 1,5 | 1,3 | 1,7 | 1,5 |
| 20 | I | 2,5 | 1,4 | ***4,5*** | 2,6 | 2,2 | 1,8 | 3,0 |
| 21 | IV | 1,0 | 1,2 | 1,3 | 1,0 | 1,3 | 1,0 | 1,2 |
| 22 | IV | 1,0 | 1,0 | 1,2 | 1,0 | 1,6 | 1,0 | 1,0 |
