# Supplementary figures and images for: Human Immunoglobulin G Cannot Inhibit Fibrinogen Binding by the Genetically Diverse A Domain of Staphylococcus aureus Fibronectin-Binding Protein A
Source: mSphere. 2018 Mar 7;3(2):e00590-17. doi: 10.1128/mSphere.00590-17 (PMC5853482; doi:10.1128/mSphere.00590-17)

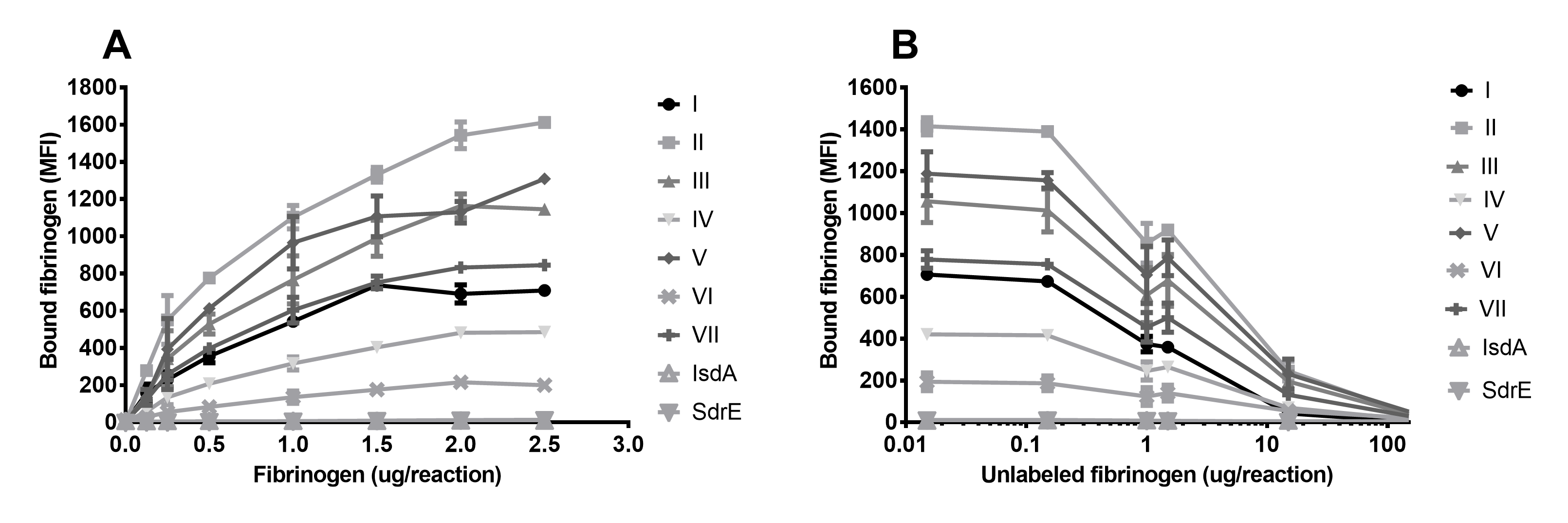

Supplement: FIG S1 [file sph002182493sf1.tif]

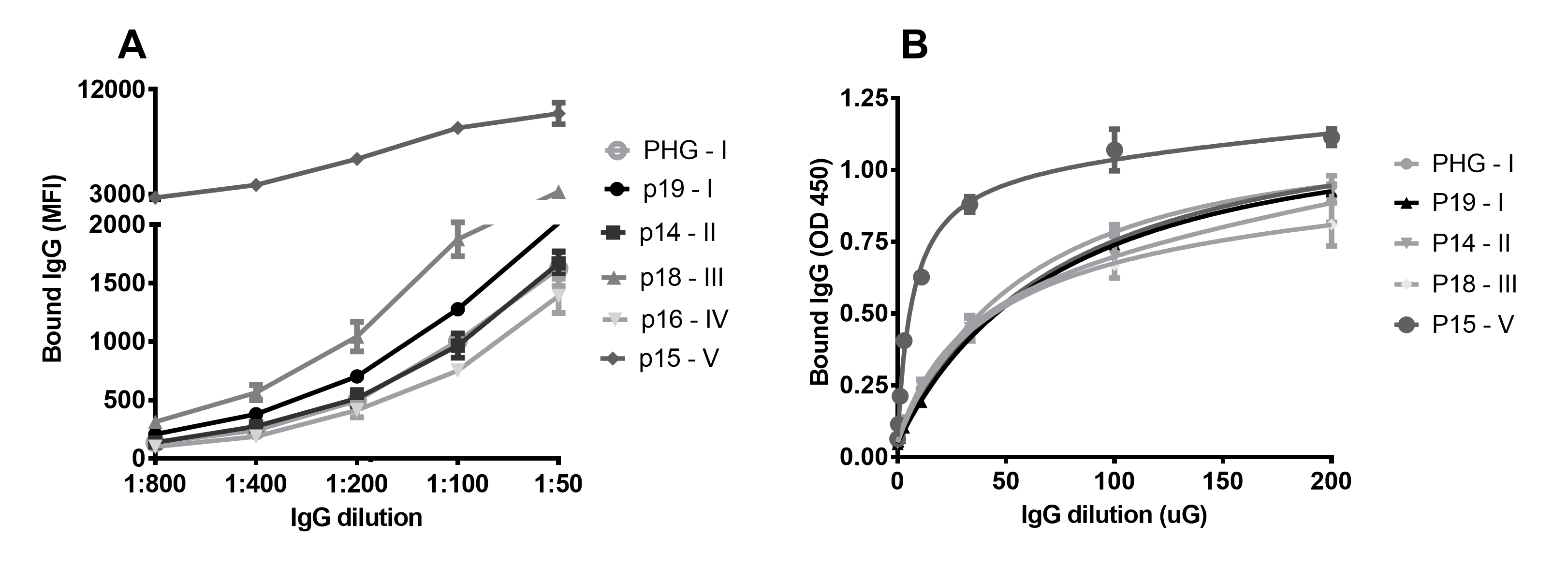

Supplement: FIG S2 [file sph002182493sf2.tif]
